# Correlation between TG/HDL-C ratio and obstructive sleep apnea in the adult US population: a cross-sectional study

**DOI:** 10.3389/fneur.2025.1594875

**Published:** 2025-06-19

**Authors:** Yanlin Yang, Gaohui Wu, Lingyun Ren

**Affiliations:** ^1^Department of Anesthesiology, Zhongnan Hospital of Wuhan University, Wuhan, China; ^2^Department of Anesthesiology, The Central Hospital of Wuhan, Wuhan, China

**Keywords:** NHANES, obstructive sleep apnea, TG/HDL-C, dyslipidemia, a cross-sectional study

## Abstract

**Background:**

Obstructive sleep apnea (OSA) is associated with dyslipidemia, neurocognitive impairment, and cardiovascular morbidity. This study investigates the triglyceride-to-high-density lipoprotein cholesterol ratio (TG/HDL-C) as a composite biomarker for OSA prevalence, with implications for mitigating OSA-associated complications through metabolic modulation.

**Methods:**

A nationally representative cohort of 3,270 adults (NHANES 2015–2018) was analyzed using weighted multivariate logistic regression. Restricted cubic splines (RCS) modeled nonlinear associations, while receiver operating characteristic (ROC) curves evaluated predictive performance against conventional lipid indices (TG, HDL-C) and composite lipid indices (TG/HDL-C, NHHR). The DeLong test further compares the predictive efficacy between models. The differences between groups in predicting OSA based on TG/HDL-C levels were further examined via subgroup analysis.

**Results:**

Significant differences in lipid profiles were observed between OSA and non-OSA cohorts (*p* < 0.001). Each unit increase in TG/HDL-C conferred a 17% elevated OSA risk [OR = 1.17; 95% CI: 1.00–1.35; *p* = 0.045], with a nonlinear dose–response relationship (*p* for interaction = 0.017). TG/HDL-C demonstrated superior predictive accuracy (AUC = 0.589) versus NHHR (AUC = 0.572). Subgroup analyses indicated that TG/HDL-C as an indicator of OSA varied by the family income to poverty ratio (PIR) group, as well as among congestive heart failure and angina populations (*p* for interaction < 0.05).

**Conclusion:**

This study demonstrates that the TG/HDL-C ratio is a superior predictor of OSA risk compared to TG and NHHR. These findings underscore the clinical utility of composite lipid indices TG/HDL-C for early OSA detection and targeted intervention.

## 1 Introduction

Obstructive sleep apnea (OSA) is characterized by intermittent upper airway collapse during sleep, leading to transient hypopnea or apnea, consequent hypoxemia, hypercapnia, and sleep fragmentation (1). Clinically, OSA manifests as snoring, excessive daytime sleepiness, neurocognitive impairment, and erectile dysfunction, frequently comorbid with obesity and hypertension (2). Epidemiological data indicate a prevalence of 22% in males and 17% in females [apnea-hypopnea index (AHI) ≥ 5], with a significantly higher incidence observed in men (3). Notably, emerging evidence associates OSA with increased cardiovascular mortality (4). Furthermore, OSA has been implicated in various neurological disorders, including depression, attentional deficits, executive dysfunction, and verbal memory impairment (5). Given the paucity of reliable lipid biomarkers for OSA prediction, this study aims to identify and optimize lipid-based indices to predict OSA onset and mitigate its associated comorbidities.

Lipid metabolic dysregulation has been well-documented in OSA patients, marked by elevated low-density lipoprotein cholesterol (LDL), triglycerides (TG), and total cholesterol (TC), alongside reduced high-density lipoprotein cholesterol (HDL-C) (6, 7). These aberrations constitute independent risk factors for cardiovascular disease (8). A retrospective clinical analysis revealed a positive correlation between the TG/HDL-C ratio and OSA severity (9). Mechanistically, OSA-induced hyperlipidemia may arise from impaired lipoprotein clearance due to suppressed lipoprotein lipase (LpL) activity. Preclinical studies demonstrate that prolonged intermittent hypoxia inhibits triglyceride-rich lipoprotein (TRLP) catabolism, resulting in elevated TC and TG (10). Additionally, murine models of acute hypoxia exhibit downregulated PPAR-γ expression—a key regulator of lipid metabolism—leading to reduced LpL activity and consequent dyslipidemia (11).

Patients with OSA typically present with disorders of lipid metabolism, which include abnormalities of TG/HDL-C (9). Changes in TG/HDL-C values can cause a number of health problems. According to prior research, the TG/HDL-C correlates with insulin resistance and identifies patients with type 2 diabetes with poor glycemic control (12). TG/HDL-C is a new indicator of metabolic syndrome and cardiovascular disease (13). TG/HDL-C levels have also been reported to affect later cognitive function during acute ischemic stroke (14). The elevation in the value of TG/HDL-C possibly enhances the likelihood of fatty liver (15).

While prior investigations have predominantly examined individual lipid parameters in OSA, the diagnostic utility of composite lipid indices remains underexplored. This study comprehensively evaluates the association between TG/HDL-C and OSA severity, positing that OSA pathogenesis is influenced by synergistic lipid abnormalities. Compared to conventional lipid measures and the NHHR, TG/HDL-C may serve as a superior cardiovascular risk biomarker in OSA patients (16). These findings could inform personalized lipid-modifying therapies to attenuate OSA-related cardiovascular and neurological sequelae.

## 2 Method

### 2.1 Study population and design

**Figure 1:**
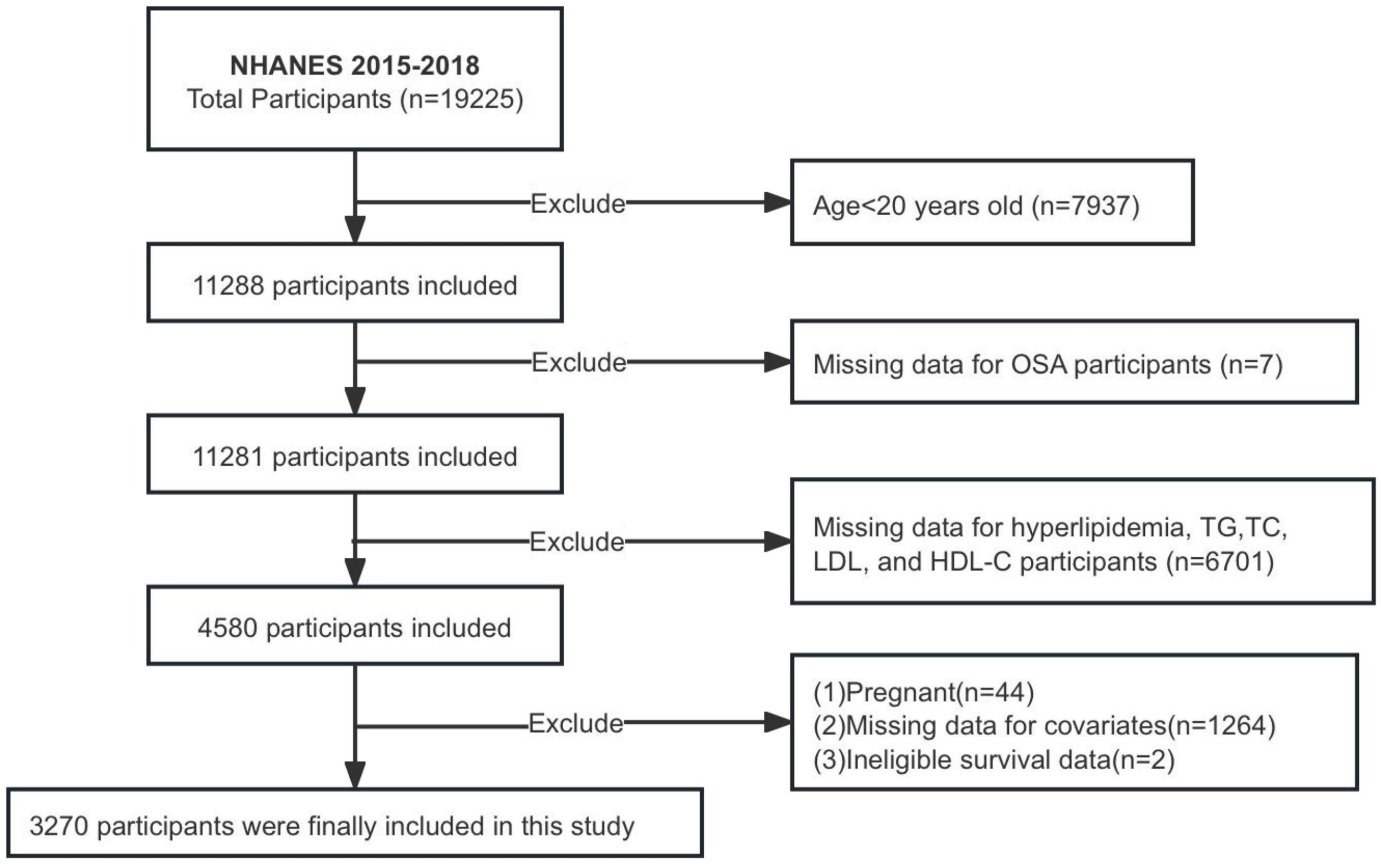
Flowchart of participants selection.

The NHANES database, which collects population data through personal interviews and standardized physical examinations, laboratory measurements, and the participation and organization of specially trained personnel, is posted on the website http://www.cdc.gov/nchs/Nhanes/ and is available to the public. Participants in the NHANES study granted informed consent, and the study received approval from the National Center for Health Statistics Research Ethics Board. This study gathered data from participants ≥20 years old between 2015 and 2018. Ultimately, 3,270 patients were enrolled in the study after removing individuals with missing data on OSA, various lipid indices, and covariates, as well as pregnant individuals and those lacking follow-up information (Figure 1).

### 2.2 Exposure variable

Data for HDL-C, LDL, TC, and TG were obtained from laboratory test results available on the NHANES website. The NHHR index was calculated as (TC-HDL-C) divided by HDL-C, and TG/HDL-C was calculated as TG divided by HDL-C. The diagnostic criteria for hyperlipidemia include: (1) hypertriglyceridemia: TG ≥ 150 mg/dL; (2) hypercholesterolemia: TC ≥ 200 mg/dL, LDL ≥ 130 mg/dL, HDL < 40 mg/dL; (3) utilization of lipid-lowering medications. At least one of these criteria must be satisfied.

### 2.3 Outcome variable

The OSA diagnosis was derived from a questionnaire on the NHANES website. The questionnaire assessed the presence of snoring, frequency of snoring or apnea episodes, and daytime sleepiness occurrences. OSA symptoms: (1) snoring ≥3 times per week at night; (2) snorting, gasping, or stopping breath ≥3 times per week at night; (3) feeling excessively sleepy during the day on 16–30 occasions per month, despite weekdays or workdays with roughly seven or additional hours of sleep each night. At least one of the three criteria is satisfied (17).

### 2.4 Covariates

NHANES gathered demographic and health-related data through questionnaires encompassing age, sex, race, PIR, education, smoking and drinking habits, hypertension, and cardiovascular disease (CVD); follow-up data (concluding December 31, 2019) were incorporated to ascertain cardiac cause mortality and all-cause mortality rates. Race is classed as white, black, mexican, and others; marital status is categorized as having a spouse or not; and educational attainment is categorized as having a high school education; smoking status was classified as never (less than 100 cigarettes), former (smoked over 100 cigarettes but is no longer smoking), and current (smoked more than 100 cigarettes and is still smoking). Alcohol consumption was classified as never, current (exceeding 12 drinks and still consuming), and former (having consumed 12 drinks in the past but not presently drinking); PIR categorized as low-income and non-low-income (<1.0, ≥1.0); patients were classed as suffering from CVD if they had any of the subsequent conditions: congestive heart failure, angina, coronary heart disease, or stroke.

### 2.5 Statistical analysis

The analysis utilized the weights supplied by the NHANES website. The chi-square and ANOVA tests were utilized in descriptive analysis. The mean ± standard error was reported for continuous variables. Categorical variables were represented as percentages. Mann–Whitney U was employed to compare several lipid indices (TG, TC, LDL, HDL-C, NHHR, TG/HDL-C) between OSA and non-OSA patients. Constructing multivariate logistic regression models incorporating different covariates (I, II, and III) to clarify the association between OSA and TG/HDL-C. Model I is unadjusted. Model II incorporates adjustment variables from Model I: sex, age, and race. Model III includes additional adjustment variables to Model II: sex, age, race, PIR, BMI, educational levels, hypertension, marital status, smoking, alcohol use, and CVD.

RCS employed logistic regression modeling to investigate the potential nonlinearity of the connection between each lipid index response (TG, TC, LDL, HDL-C, NHHR, TG/HDL-C) and OSA. ROC curves derived from logistic regression modeling, which compared the area under the curve (AUC) of TG, HDL-C, NHHR, and TG/HDL-C, were employed to predict the efficacy in assessing the risk of developing OSA. The DeLong test further compares the ROC models two by two. Finally, subgroup studies were conducted to investigate potential variations in TG/HDL-C for diagnosing OSA across different groups. Data were analyzed utilizing R (Version 4.3.1, The R Foundation, Vienna, Austria), MedCalc (Version 11.4.2.0, Ostend, Belgium), and GraphPad Prism (Version 10.0.0, San Diego, California, United States). Statistical significance is denoted by a *p* value < 0.001 or < 0.05.

## 3 Results

### 3.1 The basic traits of participants

The study included 3,270 participants (49.14% male; mean age 47.72 ± 0.56 years), with an overall OSA prevalence of 33.52%. When stratified by TG/HDL-C quartiles, participants in Quartile 4 were more likely to be older, male, current smokers, and have a lower education level, higher BMI, hypertension, hyperlipidemia, or angina compared to Quartile 1 (all *p* < 0.05). Lipid profiles differed significantly across quartiles, with Quartile 4 exhibiting elevated TC, TG, LDL, NHHR, and TG/HDL-C, alongside reduced HDL-C (*p* < 0.001) (Table 1).

**Table 1:** The characteristics of participants were stratified by TG/HDL-C, from NHANES 2015–2018.

Variable	Total	Quartiles of TG/HDL-C				*p*-value
		Q1	Q2	Q3	Q4	
Age, mean (SE)	47.72 (0.56)	45.54 (1.17)	47.20 (0.73)	49.05 (0.84)	49.19 (0.63)	0.039
Sex (%)						<0.001
Male	1,607 (49.14)	299 (35.93)	363 (45.38)	436 (54.04)	509 (62.23)	
Female	1,663 (50.86)	520 (64.07)	453 (54.62)	381 (45.96)	309 (37.77)	
Race (%)						<0.001
White	1,174 (35.90)	280 (67.33)	266 (64.08)	303 (67.49)	325 (67.98)	
Black	677 (20.70)	261 (14.84)	218 (12.86)	127 (7.65)	71 (4.19)	
Mexican	505 (15.44)	83 (5.55)	112 (7.62)	143 (9.04)	167 (11.43)	
Others	914 (27.95)	195 (12.28)	220 (15.44)	244 (15.82)	255 (16.40)	
Marital status (%)						0.014
Married/living with partner	1,973 (60.34)	443 (62.59)	476 (58.38)	519 (66.34)	535 (70.01)	
Widowed/divorced/separated/never married	1,297 (39.66)	376 (37.41)	340 (41.62)	298 (33.66)	283 (29.99)	
Education level (%)						<0.001
<High school	621 (18.99)	114 (7.88)	156 (11.36)	164 (9.92)	187 (14.30)	
Completed high school	755 (23.09)	170 (20.47)	178 (22.62)	203 (27.02)	204 (28.46)	
>High school	1,894 (57.92)	535 (71.65)	482 (66.02)	450 (63.05)	427 (57.25)	
Hypertension (%)						<0.001
No	1,868 (57.13)	542 (72.94)	495 (66.87)	411 (56.01)	420 (50.62)	
Yes	1,402 (42.87)	277 (27.06)	321 (33.13)	406 (43.99)	398 (49.38)	
Drinking (%)						0.089
No	746 (22.81)	185 (14.85)	188 (18.81)	175 (15.61)	198 (19.68)	
Yes	2,524 (77.19)	634 (85.15)	628 (81.19)	642 (84.39)	620 (80.32)	
Smoking (%)						0.004
Never	1,844 (56.39)	514 (60.14)	474 (58.95)	461 (56.63)	395 (46.13)	
Former	794 (24.28)	178 (25.98)	190 (24.58)	203 (25.15)	223 (31.22)	
Now	632 (19.33)	127 (13.87)	152 (16.48)	153 (18.22)	200 (22.66)	
PIR (%)						0.339
<1.00	634 (19.39)	145 (10.21)	169 (13.07)	160 (12.73)	160 (13.20)	
≥1.00	2,636 (80.61)	674 (89.79)	647 (86.93)	657 (87.27)	658 (86.80)	
BMI (%)						<0.001
<18.5 kg/m2	54 (1.65)	33 (3.70)	11 (1.83)	6 (0.60)	4 (0.80)	
18.5 to < 25.0 kg/m2	841 (25.72)	371 (47.11)	246 (31.01)	132 (16.71)	92 (8.74)	
25.0 to < 30.0 kg/m2	1,042 (31.87)	231 (29.83)	253 (29.96)	289 (34.58)	269 (30.91)	
≥30.0 kg/m2	1,333 (40.76)	184 (19.36)	306 (37.20)	390 (48.12)	453 (59.54)	
Congestive heart failure (%)						0.030
No	3,171 (96.97)	803 (99.04)	793 (98.57)	789 (97.43)	786 (97.11)	
Yes	99 (3.03)	16 (0.96)	23 (1.43)	28 (2.57)	32 (2.89)	
Angina (%)						0.003
No	3,197 (97.77)	808 (99.08)	805 (98.94)	793 (98.06)	791 (95.58)	
Yes	73 (2.23)	11 (0.92)	11 (1.06)	24 (1.94)	27 (4.42)	
Coronary heart disease (%)						0.105
No	3,139 (95.99)	794 (96.76)	788 (97.94)	780 (96.40)	777 (94.74)	
Yes	131 (4.01)	25 (3.24)	28 (2.06)	37 (3.60)	41 (5.26)	
Stroke (%)						0.280
No	3,155 (96.48)	795 (97.98)	787 (97.70)	788 (97.91)	785 (96.51)	
Yes	115 (3.52)	24 (2.02)	29 (2.30)	29 (2.09)	33 (3.49)	
CVD (%)						0.004
No	2,976 (91.01)	768 (94.79)	747 (94.27)	737 (92.81)	724 (89.29)	
Yes	294 (8.99)	51 (5.21)	69 (5.73)	80 (7.19)	94 (10.71)	
Hyperlipidemia (%)						<0.001
No	1,005 (30.73)	453 (58.82)	337 (42.13)	196 (24.55)	19 (3.51)	
Yes	2,265 (69.27)	366 (41.18)	479 (57.87)	621 (75.45)	799 (96.49)	
TG (mmol/L), mean (SE)	1.38 (0.02)	0.69 (0.01)	1.01 (0.01)	1.41 (0.01)	2.42 (0.04)	<0.001
TC (mmol/L), mean (SE)	4.90 (0.03)	4.67 (0.05)	4.75 (0.04)	4.94 (0.05)	5.22 (0.04)	<0.001
LDL (mmol/L), mean (SE)	2.90 (0.02)	2.53 (0.04)	2.86 (0.03)	3.07 (0.04)	3.16 (0.03)	<0.001
HDL -C (mmol/L), mean (SE)	1.44 (0.02)	1.88 (0.02)	1.50 (0.02)	1.29 (0.01)	1.06 (0.01)	<0.001
NHHR, mean (SE)	2.67 (0.03)	1.56 (0.02)	2.23 (0.03)	2.87 (0.03)	4.06 (0.06)	<0.001
TG/HDL-C, mean (SE)	1.13 (0.02)	0.38 (0.00)	0.68 (0.00)	1.10 (0.01)	2.39 (0.05)	<0.001
Cardiac mortality (%)						0.657
No	3,243 (99.17)	815 (99.70)	810 (99.48)	807 (99.28)	811 (99.15)	
Yes	27 (0.83)	4 (0.30)	6 (0.52)	10 (0.72)	7 (0.85)	
All-cause mortality (%)						0.618
No	3,168 (96.88)	796 (98.15)	781 (97.09)	794 (98.05)	797 (97.56)	
Yes	102 (3.12)	23 (1.85)	35 (2.91)	23 (1.95)	21 (2.44)	
OSA (%)						<0.001
No	2,174 (66.48)	615 (78.57)	560 (69.09)	531 (66.04)	468 (56.01)	
Yes	1,096 (33.52)	204 (21.43)	256 (30.91)	286 (33.96)	350 (43.99)	

SE, standard error; PIR, family income to poverty ratio; BMI, body mass index; CVD, cardiovascular disease; OSA, obstructive sleep apnea; TG, triglyceride; TC, total cholesterol; HDL-C, high-density lipoprotein cholesterol; LDL, low-density lipoprotein; NHHR, non-high-density lipoprotein cholesterol to high-density lipoprotein cholesterol ratio; TG/HDL-C, triglyceride to high-density lipoprotein cholesterol ratio.

Continuous variables were described as mean (standard error), and categorical variables were expressed as numbers (percentage). *p* values were calculated by weight χ^2^ test or Wilcoxon.

**Figure 2:**
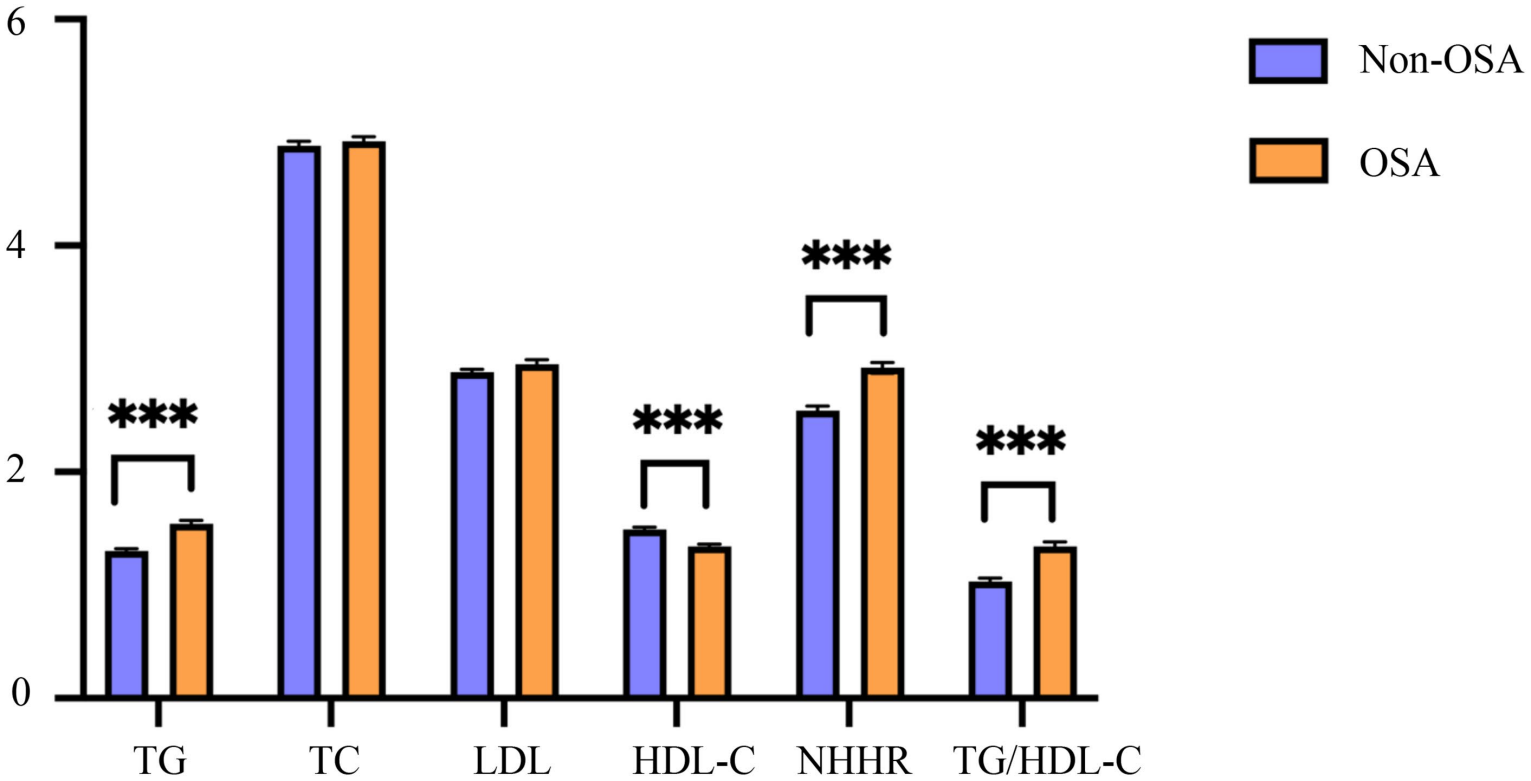
Characteristics of blood lipid indexes in the OSA and the non-OSA group. OSA, obstructive sleep apnea; Non-OSA, without obstructive sleep apnea; TG, triglyceride; TC, total cholesterol; HDL-C, high-density lipoprotein cholesterol; LDL, low-density lipoprotein; NHHR, non-high-density lipoprotein cholesterol to high-density lipoprotein cholesterol ratio; TG/HDL-C, triglyceride to high-density lipoprotein cholesterol ratio. ***p < 0.001.

Comparative analysis revealed significant differences in lipid parameters between OSA and non-OSA groups (Figure 2). The OSA cohort demonstrated higher mean values of TG, NHHR, and TG/HDL-C, but lower HDL-C (all *p* < 0.001).

### 3.2 Relationship between TG/HDL-C, NHHR, TC, TG, LDL, HDL-C, and OSA prevalence

The results of multivariate logistic regression indicated a positive linear connection between TG/HDL-C, TG, HDL-C, and the incidence of OSA. Table 2 demonstrates that, after controlling for all covariates (sex, age, race, PIR, BMI, education levels, hypertension, marital status, smoking, drinking, and CVD), the incidence of OSA increased by 17% with each unit increase in TG/HDL-C [OR = 1.17; 95% CI: 1.00–1.35; *p* = 0.045]; the prevalence of OSA rose by 18% with each unit rise in TG [OR = 1.18; 95% CI: 1.01–1.38; *p* = 0.035], and the prevalence of OSA diminished by 38% with each unit elevation in HDL-C [OR = 0.62; 95% CI: 0.45–0.85; *p* = 0.006]. In addition, quartile 4 of TG/HDL-C exhibited a 69% increased probability of OSA in comparison to quartile 1 of TG/HDL-C [OR = 1.69; 95% CI: 1.17–2.43; *p* = 0.010].

**Table 2:** Association between TG/HDL-C and OSA prevalence in the total population.

Outcomes	Model I		Model II		Model III	
	OR (95%CI)	*P*	OR (95%CI)	*P*	OR (95%CI)	*P*
TG/HDL-C	1.41 (1.24, 1.61)	<0.001	1.39 (1.21, 1.60)	<0.001	1.17 (1.00, 1.35)	0.045
NHHR	1.27 (1.15, 1.39)	<0.001	1.26 (1.14, 1.40)	<0.001	1.11 (0.99, 1.24)	0.077
TC	1.03 (0.94, 1.13)	0.523	1.01 (0.93, 1.10)	0.808	0.99 (0.89, 1.11)	0.921
TG	1.47 (1.29, 1.67)	<0.001	1.43 (1.24, 1.64)	<0.001	1.18 (1.01, 1.38)	0.035
LDL	1.09 (0.98, 1.21)	0.118	1.07 (0.96, 1.19)	0.192	1.02 (0.90, 1.16)	0.743
HDL-C	0.44 (0.33, 0.58)	<0.001	0.40 (0.29, 0.55)	<0.001	0.62 (0.45, 0.85)	0.006
Quartile of TG/HDL-C						
Q1	ref		ref		ref	
Q2	1.64 (1.17, 2.29)	0.005	1.59 (1.14, 2.24)	0.009	1.34 (0.90, 1.99)	0.130
Q3	1.89 (1.30, 2.73)	0.002	1.80 (1.24, 2.62)	0.004	1.27 (0.85, 1.88)	0.210
Q4	2.88 (2.10, 3.95)	<0.001	2.75 (1.96, 3.85)	<0.001	1.69 (1.17, 2.43)	0.010
*P* for trend		<0.001		<0.001		0.011

Model I, not adjusted.

Model II, adjustment variables were added to Model I: sex, age, and race.

Model III, adjustment variables were added to Model II: sex, age, race, PIR, BMI, education levels, hypertension, marital status, smoking, drinking, and CVD.

OR, odds ratio; CI, confidence interval; PIR, family income to poverty ratio; BMI, body mass index; CVD, cardiovascular disease; OSA, obstructive sleep apnea; TG, triglyceride; TC, total cholesterol; HDL-C, high-density lipoprotein cholesterol; LDL, low-density lipoprotein; NHHR, non-high-density lipoprotein cholesterol to high-density lipoprotein cholesterol ratio; TG/HDL-C, triglyceride to high-density lipoprotein cholesterol ratio.

### 3.3 RCS revealed the nonlinear relationship between TG/HDL-C, NHHR, TC, TG, LDL, HDL-C, and OSA prevalence

**Figure 3:**
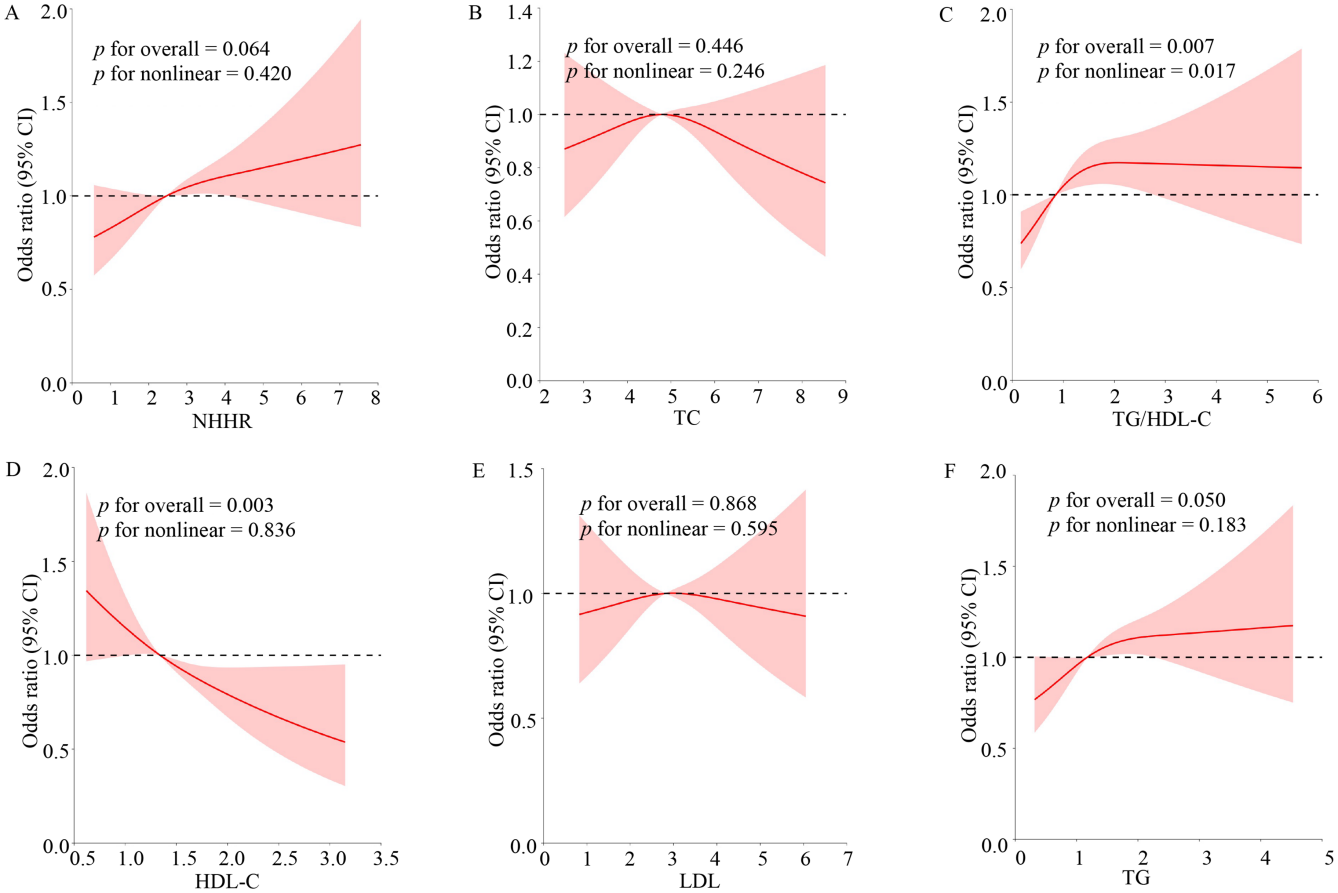
Non-linear relationship of blood lipid indexes and OSA. The model adjusted the variables for sex, age, race, PIR, BMI, education levels, hypertension, marital status, smoking, drinking, and CVD. The solid red lines indicated the prevalence of OSA, 95% of CIs were applied to the figure. OR, odds ratio; 95% of CI, 95% confidence interval; OSA, obstructive sleep apnea; TG, triglyceride; TC, total cholesterol; HDL-C, high-density lipoprotein cholesterol; LDL, low-density lipoprotein; NHHR, non-high-density lipoprotein cholesterol to high-density lipoprotein cholesterol ratio; TG/HDL-C, triglyceride to high-density lipoprotein cholesterol ratio. **(A–F)** Stands for image encoding.

RCSs were employed to study the nonlinear connection between TG/HDL-C, NHHR, TC, TG, LDL, HDL-C, and OSA disease (Figure 3). After correcting for all factors in the multivariate logistic regression model III above, a nonlinear correlation existed between TG/HDL-C and the OSA group (*p* for overall = 0.007; *p* for nonlinear = 0.017). TG and HDL-C were associated with the incidence of OSA, but there was no nonlinear relationship (*p* for overall ≤ 0.05; *p* for nonlinear > 0.05).

### 3.4 ROC curves combined with the DeLong test to predict OSA

**Figure 4:**
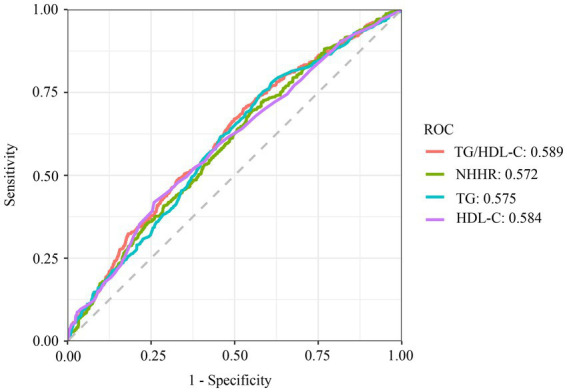
ROC curve analysis of TG, HDL-C, NHHR, TG/HDL-C. TG, triglyceride; TC, total cholesterol; HDL-C, high-density lipoprotein cholesterol; LDL, low-density lipoprotein; NHHR, non-high-density lipoprotein cholesterol to high-density lipoprotein cholesterol ratio; TG/HDL-C, triglyceride to high-density lipoprotein cholesterol ratio; ROC, receiver operating characteristic.

The result demonstrated TG/HDL-C’s superior discriminative capacity for OSA (AUC = 0.589; sensitivity = 67.6%, specificity = 46.4%) compared to NHHR (AUC = 0.572) and TG (AUC = 0.575) (Figure 4). However, there was no statistical difference between ROC models for TG/HDL-C and HDL-C metrics to predict the prevalence of OSA (*p* > 0.05) (Supplementary material).

### 3.5 Subgroup analysis for TG/HDL-C

After adjusting for covariates (sex, age, race, PIR, BMI, education levels, hypertension, marital status, smoking, drinking, and CVD), multi-subgroup analyses and interaction tests were performed to assess the reliability of using TG/HDL-C to diagnose the OSA population and to identify potential population differences (Table 3). The correlation between TG/HDL-C and OSA was consistent across most categories. However, congestive heart failure (*p* for interaction = 0.013), angina (*p* for interaction = 0.016), and PIR (*p* for interaction = 0.006) groups altered this association. The TG/HDL-C diagnosis of OSA was more applicable to the group without angina [OR = 1.11; 95% CI:1.02–1.20; *p* = 0.019], the group without congestive heart failure [OR = 1.11; 95% CI:1.02–1.21; *p* = 0.017], and the group with PIR greater than or equal to 1.00 [OR = 1.18; 95% CI:1.07–1.30; *p* < 0.001].

**Table 3:** Subgroup analysis for the connection between TG/HDL-C and OSA.

Variables	OSA (*n*%)	OR (95%CI)	*P*	*P* for interaction
All patients	3,270 (100.00)	1.09 (1.00 ~ 1.18)	0.043	
Sex				0.122
Male	1,607 (49.14)	1.04 (0.94 ~ 1.14)	0.465	
Female	1,663 (50.86)	1.22 (1.05 ~ 1.41)	0.011	
Race				0.424
White	1,174 (35.90)	1.10 (0.95 ~ 1.27)	0.207	
Black	677 (20.70)	1.39 (1.07 ~ 1.80)	0.013	
Mexican	505 (15.44)	1.06 (0.91 ~ 1.24)	0.463	
Others	914 (27.95)	1.02 (0.86 ~ 1.20)	0.826	
Marital status				0.647
Married/living with partner	1,973 (60.34)	1.08 (0.98 ~ 1.19)	0.132	
Widowed/divorced/separated/never married	1,297 (39.66)	1.11 (0.95 ~ 1.29)	0.188	
Education level				0.375
<High school	621 (18.99)	1.04 (0.90 ~ 1.20)	0.603	
Completed high school	755 (23.09)	1.01 (0.84 ~ 1.23)	0.896	
>High school	1,894 (57.92)	1.16 (1.03 ~ 1.30)	0.014	
Hypertension				0.741
No	1,868 (57.13)	1.10 (0.98 ~ 1.24)	0.115	
Yes	1,402 (42.87)	1.07 (0.95 ~ 1.19)	0.270	
Drinking				0.823
No	746 (22.81)	1.07 (0.89 ~ 1.30)	0.458	
Yes	2,524 (77.19)	1.08 (0.99 ~ 1.19)	0.086	
Smoking				0.939
Never	1,844 (56.39)	1.09 (0.96 ~ 1.24)	0.174	
Former	794 (24.28)	1.10 (0.96 ~ 1.27)	0.167	
Now	632 (19.33)	1.08 (0.90 ~ 1.29)	0.439	
PIR				0.006
<1.00	634 (19.39)	0.88 (0.72 ~ 1.07)	0.190	
≥1.00	2,636 (80.61)	1.18 (1.07 ~ 1.30)	<0.001	
BMI				0.605
<18.5 kg/m2	54 (1.65)	0.21 (0.01 ~ 8.94)	0.418	
18.5 to < 25.0 kg/m2	841 (25.72)	1.09 (0.81 ~ 1.46)	0.577	
25.0 to < 30.0 kg/m2	1,042 (31.87)	0.99 (0.86 ~ 1.15)	0.933	
≥30.0 kg/m2	1,333 (40.76)	1.16 (1.04 ~ 1.30)	0.010	
Congestive heart failure				0.013
No	3,171 (96.97)	1.11 (1.02 ~ 1.21)	0.017	
Yes	99 (3.03)	0.66 (0.40 ~ 1.08)	0.099	
Angina				0.016
No	3,197 (97.77)	1.11 (1.02 ~ 1.20)	0.019	
Yes	73 (2.23)	0.52 (0.27 ~ 1.02)	0.058	
Coronary heart disease				0.200
No	3,139 (95.99)	1.10 (1.01 ~ 1.19)	0.035	
Yes	131 (4.01)	0.90 (0.59 ~ 1.36)	0.607	
Stroke				0.987
No	3,155 (96.48)	1.09 (1.01 ~ 1.19)	0.036	
Yes	115 (3.52)	0.89 (0.51 ~ 1.57)	0.687	
CVD				0.098
No	2,976 (91.01)	1.11 (1.02 ~ 1.22)	0.017	
Yes	294 (8.99)	0.90 (0.68 ~ 1.21)	0.500	
Hyperlipidemia				0.520
No	1,005 (30.73)	1.26 (0.74 ~ 2.13)	0.397	
Yes	2,265 (69.27)	1.05 (0.96 ~ 1.15)	0.292	

Covariates: sex, age, race, PIR, BMI, education levels, hypertension, marital status, smoking, drinking, and CVD.

OR, odds ratio; CI, confidence interval; PIR, family income to poverty ratio; BMI, body mass index; CVD, cardiovascular disease; OSA, obstructive sleep apnea; TG, triglyceride; TC, total cholesterol; HDL-C, high-density lipoprotein cholesterol; LDL, low-density lipoprotein; NHHR, non-high-density lipoprotein cholesterol to high-density lipoprotein cholesterol ratio; TG/HDL-C, triglyceride to high-density lipoprotein cholesterol ratio.

## 4 Discussion

This large-scale cross-sectional study elucidated the association between the composite lipid marker TG/HDL-C and the incidence of OSA. TG/HDL-C was identified as an independent risk factor for OSA development [OR = 1.17; 95% CI: 1.00–1.35; *p* = 0.045]. Notably, elevated TG/HDL-C levels demonstrated a stronger association with OSA prevalence than individual lipid markers or NHHR. RCS regression revealed a nonlinear dose–response relationship between TG/HDL-C levels and OSA (*p* for nonlinear < 0.05). TG/HDL-C exhibited superior discriminative accuracy for OSA compared to TG and NHHR (AUC: 0.589 vs. 0.572–0.575). Furthermore, the relationship between TG/HDL-C and OSA varied by PIR, congestive heart failure, and angina. In conclusion, this study is an independent association study of the composite lipid index TG/HDL-C with OSA disease.

Numerous studies have established a link between OSA and dyslipidemia. However, the extant literature has predominantly focused on individual lipid parameters, overlooking the potential clinical utility of composite lipid indices. A study found that OSA patients had markedly elevated lipid levels (TG, TC, LDL) compared to healthy individuals (18). Supporting this observation, a prospective cohort study involving 93 persons further established positive correlations between TG, TC, and LDL and OSA severity (19). Notably, Barceló et al. conducted a targeted investigation, isolating LDL particles from 14 severe OSA patients and 13 matched controls. Their findings revealed a pronounced increase in thiobarbituric acid-reactive substances (TBARS) and aberrant lipid peroxidation profiles among OSA patients, suggesting that oxidative stress may underlie the dyslipidemia-OSA interplay (20).

While substantial evidence links dyslipidemia to OSA, notable inconsistencies exist across studies. A large cross-sectional analysis (*n* = 753 males) failed to demonstrate significant associations between OSA and conventional lipid parameters (TG, HDL-C, LDL-C, TC; all *p* > 0.05 after multivariable adjustment) (21). This null finding may reflect population homogenization, as shared regional lifestyle and dietary patterns among participants could attenuate true biological associations. Further complicating the evidence base, a case–control investigation (*n* = 32 OSA patients) reported no correlation between severe OSA and LDL, HDL-C levels (22). The clinical interpretation of these results is constrained by methodological limitations, including inadequate sample size and the absence of a matched control group. These discrepant findings underscore the critical importance of considering population characteristics and study design when evaluating the lipid-OSA relationship.

This study identified significant linear associations between OSA and multiple lipid indices, including TG/HDL-C ratio [OR = 1.17; 95% CI: 1.00–1.35; *p* = 0.045], TG [OR = 1.18; 95% CI: 1.01–1.38; *p* = 0.035], and HDL-C [OR = 0.62; 95% CI: 0.45–0.85; *p* = 0.006]. These findings align with established pathophysiological mechanisms linking dyslipidemia to OSA. Experimental evidence from murine models demonstrates that high-fat diet-induced obesity downregulates PPAR-γ expression, triggering inflammatory cascades and oxidative stress that potentiate chronic intermittent hypoxia (23). Oxidative stress produces dysfunctional oxidized lipids while decreasing HDL-C levels through impaired reverse cholesterol transport (24). In addition, OSA-induced hypoxia activates the sympathetic nervous system (25), resulting in complex regulation of lipid metabolism. Inhibition of both beta and alpha receptors influences lipid profiles: specifically, antagonism of alpha-1 receptors elevates HDL levels and reduces blood triglycerides, whereas blockade of beta-adrenergic receptors yields contrary effects (26–28). These interacting mechanisms may explain the association between sympathetic overactivity and atherogenic dyslipidemia observed in patients with OSA.

ROC analysis and DeLong test demonstrated superior predictive performance of TG/HDL-C for OSA compared to conventional lipid markers and NHHR. This finding gains biological plausibility from genome-wide analyses identifying TG/HDL-C as a surrogate marker for insulin resistance (29), a metabolic derangement frequently comorbid with OSA (30–32). Louis et al. identified a study that subjected healthy individuals to intermittent hypoxia for 5 h, resulting in a decline in insulin sensitivity (33). Another study, selecting young healthy adults to selectively inhibit slow-wave sleep (SWS) at night without modifying overall sleep length, led to impaired insulin sensitivity and decreased glucose tolerance (34).

Subgroup analyses showed that the association of TG/HDL with OSA was more applicable to non-poor populations (PIR ≥ 1.00) without congestive heart failure and angina. The relationship between lipids and OSA in impoverished populations is likely to be affected by the BMI. Congestive heart failure and angina populations may develop OSA caused by extreme hypoxemia (35). In patients with severe OSA, fluctuations in lipid levels did not produce statistical differences (22).

The findings of this study demonstrate a high degree of reliability, as the conclusions align with prior research examining the relationship between OSA and dyslipidemia. The substantial sample size (*n* = 3,270) provides sufficient statistical power to support the observed correlation. However, several limitations must be acknowledged. First, the cross-sectional design precludes causal inference, and residual confounding may persist despite multivariate adjustments. OSA and dyslipidemia may exhibit a bidirectional relationship. For instance, patients with OSA may develop dyslipidemia, while pre-existing dyslipidemia beyond a certain threshold could potentially contribute to the onset or exacerbation of OSA. Second, OSA diagnosis relied on self-reported questionnaires, introducing potential response and recall biases that could reduce the specificity of ROC analyses. Incorporating polysomnography (PSG) in future studies would enhance diagnostic accuracy and improve generalizability. Third, the absence of long-term follow-up data limited our ability to assess survival outcomes, particularly cardiovascular mortality. A prospective cohort design with extended follow-up is warranted to validate these findings. Fourth, the interpretation of subgroup analyses for congestive heart failure and angina may be constrained by limited sample sizes. Given the relatively low global prevalence of these conditions—1–3% for heart failure (36) and 3.8% for myocardial infarction (37)—the statistical power of these comparisons was inherently restricted. Notably, in this study, CVD encompasses four major events (congestive heart failure, angina, coronary heart disease, and stroke), which collectively determine its clinical heterogeneity. Therefore, the observed relationships between the TG/HDL-C and OSA need to be interpreted in the broader context of composite CVD outcomes rather than isolated subgroups. Finally, the study population was restricted to U. S. adults; thus, external validation in diverse ethnic and geographic cohorts is necessary to ensure broader applicability.

The results of this research demonstrated the superiority of TG/HDL-C as a biomarker to predict OSA risk in adults compared to previous NHHR index predictions. The results of this study have therapeutic value, potentially allowing clinicians to apply early therapies for dyslipidemia as a predictor of OSA and reduce the likelihood of its development. This composite index is more sensitive to the balance of the patient’s blood lipids than the former single index and serves as a valuable reference for guiding therapeutic medication utilization. This study establishes the TG/HDL-C as a superior biomarker for predicting OSA risk in adult populations compared to the single lipid index and NHHR. The findings hold significant clinical relevance, as early identification of dyslipidemia patterns through TG/HDL-C monitoring may enable preemptive therapeutic interventions to mitigate OSA progression. Notably, the TG/HDL-C ratio demonstrates enhanced sensitivity and specificity in detecting lipid metabolic imbalances compared to single-parameter indices. This composite biomarker provides a more physiological representation of atherogenic dyslipidemia, thereby offering clinicians a valuable tool for risk stratification, therapeutic decision-making, and monitoring treatment efficacy in at-risk populations.

## 5 Conclusion

This study investigated the association between the TG/HDL-C and OSA risk. Multivariate logistic regression revealed that each unit increment in TG/HDL-C was independently associated with a 17% elevated OSA risk [OR = 1.17; 95% CI: 1.00–1.35; *p* = 0.045]. ROC analysis and DeLong test demonstrated superior predictive performance of TG/HDL-C compared to TG and NHHR. The results suggest that modulation of lipid homeostasis through TG/HDL-C monitoring could serve as a preventive strategy for healthcare providers to mitigate OSA incidence. These findings have important clinical implications for early OSA risk stratification in at-risk populations, personalized therapeutic approaches targeting lipid metabolism, and potential reduction of OSA-related cardiovascular and neurological complications. However, the reliance on questionnaire-based OSA diagnosis in our study population may introduce detection bias, potentially affecting the observed associations. Future studies incorporating polysomnography-confirmed diagnoses are warranted to validate these findings.

## Data Availability

Publicly available datasets were analyzed in this study. This data can be found here: https://www.cdc.gov/nchs/nhanes/index.html.
