# Supplementary material for: Correlation between TG/HDL-C ratio and obstructive sleep apnea in the adult US population: a cross-sectional study
Source: Front Neurol. 2025 Jun 19;16:1594875. doi: 10.3389/fneur.2025.1594875 (PMC12221937; doi:10.3389/fneur.2025.1594875)
Supplement: Supplementary file 1 [file Table_1.docx]

The DeLong test between TG/HDL-C, NHHR, TG, and HDL-C models.

Abbreviations: TG: triglyceride; HDL-C: high-density lipoprotein cholesterol; NHHR: non-high-density lipoprotein cholesterol to high-density lipoprotein cholesterol ratio; TG/HDL-C: triglyceride to high-density lipoprotein cholesterol ratio.

| Variable | Difference between areas | Standard Error | 95% Confidence Interval | z statistic | *p* value |  |
| --- | --- | --- | --- | --- | --- | --- |
|  |  |  |  |  |  |  |
| TG/HDL-C vs NHHR | 0.0170 | 0.00687 | (0.00355,0.0305) | 2.477 | 0.0133 |  |
| TG/HDL-C vs TG | 0.0143 | 0.00387 | (0.00668,0.0218) | 3.685 | 0.0002 |  |
| TG/HDL-C vs HDL-C | 0.00498 | 0.00719 | (-0.00911,0.0191) | 0.693 | 0.4883 |  |
| NHHR vs TG | 0.00275 | 0.00869 | (-0.0143,0.0198) | 0.316 | 0.7519 |  |
| NHHR vs HDL-C | 0.0120 | 0.00758 | (-0.00282,0.0269) | 1.587 | 0.1125 |  |
| TG vs HDL-C | 0.00928 | 0.0105 | (-0.0113,0.0299) | 0.883 | 0.3775 |  |
